# Analysis of autonomic nervous function and associated symptoms in patients with peripheral vestibular disorders

**DOI:** 10.3389/fneur.2025.1660277

**Published:** 2025-10-08

**Authors:** Chuangwei Wang, Sai Zhang, Peifan Xie, Yilin Lang, Yongci Hao, Wenting Wang, Ping Gu

**Affiliations:** Department of Neurology, The First Hospital of Hebei Medical University, Shijiazhuang, China

**Keywords:** vertigo, dizziness, heart rate variability, autonomic nervous function, vestibular function

## Abstract

**Objective:**

This study aimed to investigate the differential effects of vestibular lesion sites, specifically the semicircular canals and otolith organs, on autonomic nervous system function, emotional state, and sleep quality by analyzing heart rate variability (HRV) and clinical symptom scale scores in patients with peripheral vestibular disorders.

**Methods:**

A total of 144 patients with peripheral vestibular disorders admitted between September 2023 and December 2024 were enrolled and divided into four groups based on vestibular function test results: Group A (normal semicircular canal and otolith function), Group B (abnormal otolith function), Group C (abnormal semicircular canal function), and Group D (abnormal semicircular canal and otolith function). Baseline characteristics, clinical symptoms, sleep and emotion scale scores, and HRV parameters were compared across groups.

**Results:**

Significant differences were observed in the Dizziness Handicap Inventory (DHI) total score, DHI physical and functional sub-scores, Pittsburgh Sleep Quality Index score, and HRV across the four groups (all *p* < 0.05). Standard deviation of NN intervals (SDNN) was negatively correlated with age, DHI, Hamilton Depression Rating Scale scores, and corrected QT interval (all *p* < 0.05). DHI was identified as an independent risk factor for reduced SDNN (*p* < 0.05).

**Conclusion:**

Multisite vestibular lesions significantly exacerbated functional impairment and autonomic dysfunction, underscoring the need for an integrated assessment of vestibular function, emotional state, and sleep quality for clinical management.

## 1 Introduction

Dizziness and vertigo are common clinical symptoms, affecting approximately 15–35% of individuals at least once in their lifetime (1, 2). They are often accompanied by varying autonomic symptoms, such as nausea, vomiting, emotional disturbances, and insomnia, which result from the crucial role played by vestibulo-autonomic reflexes. Vestibular stimulation-mediated autonomic function modulation primarily relies on direct and indirect neural connections between vestibular nuclei and autonomic centers in the brainstem (3). Notably, the former exert bidirectional control over sympathetic and parasympathetic nerve activity by projecting to key regions such as the dorsal motor nucleus of the vagus and the nucleus tractus solitarius, thereby influencing physiological indicators, including heart rate and blood pressure. This regulatory pathway involves complex interactions between vestibular labyrinthine afferent signals and autonomic reflexes, being crucial in cardiovascular and respiratory adjustments during postural changes (4, 5). Excessive sympathetic excitation promotes elevated catecholamine levels, further enhancing activity in limbic structures, such as the amygdala and hypothalamus, and in higher-order autonomic regulatory structures, thereby forming a feedback loop with the vestibular system. These neuroendocrine changes exacerbate vestibular symptoms (e.g., nausea, vomiting) and may also affect vestibular signal processing through central integration mechanisms, creating a vicious cycle. Nonetheless, although numerous methods for assessing autonomic nervous function exist, traditional approaches, such as plasma catecholamine testing and sympathetic skin response, present notable limitations. Most of these methods can only detect changes in the balance between sympathetic and parasympathetic activity; thus, they are not widely adopted in clinical practice (6). Therefore, heart rate variability (HRV) has gradually gained attention as a non-invasive assessment method; specifically, it refers to the beat-to-beat variation in the sinus rhythm of a subject, using the RR intervals of consecutive heartbeats as the basis for analysis and quantifying the differences between each RR interval (7). Notably, the HRV reflects the balance of the autonomic nervous system and can evaluate the activity of both sympathetic and parasympathetic nerves (8). Consequently, it is widely regarded as a biomarker for regulatory processes within the autonomic nervous system (9).

Yamada et al. (10) evaluated HRV in 17 patients with Meniere’s disease and found differences in sympathetic and parasympathetic activity between the remission and acute phases. Zhao et al. (11) monitored HRV in 48 patients with vestibular migraine and discovered autonomic dysfunction characterized by sympathetic hyperactivity and vagal underactivity. Although existing studies have explored the relationship between vestibular stimulation and the autonomic nervous system responses (3, 12), systematic investigation is lacking on how different vestibular lesions affect HRV and their association with clinical symptoms.

Therefore, this study aimed to investigate the relationship between vestibular function and autonomic regulation, sleep quality, and emotional state by analyzing HRV and related clinical scale scores in patients with different vestibular lesions. We hypothesized that patients with combined dysfunction of both the semicircular canals and otolith organs would exhibit poorer autonomic nervous function, more severe emotional disturbances, and worse sleep quality compared to those with normal vestibular function or isolated damage to a single vestibular site.

## 2 Materials and methods

### 2.1 Participants

We retrospectively enrolled 144 patients with peripheral vestibular disorders who were hospitalized at the Department of Neurology, First Hospital of Hebei Medical University, between September 2023 and December 2024.

Inclusion criteria:

1. Patients who met the diagnostic criteria for peripheral vestibular disorders with complaints of dizziness or vertigo; and.
2. Patients who signed informed consent.

Exclusion criteria: Patients with-

1. Severe cognitive impairment or psychiatric disorder;
2. Acute cerebral infarction or cerebral hemorrhage;
3. Severe cardiac arrhythmias; and.
4. Inability to cooperate with examinations.

This study was approved by the Ethics Committee of the First Hospital of Hebei Medical University, and all participants provided written informed consent.

### 2.2 General data collection

The patient’s past outpatient, emergency, and inpatient medical records were reviewed. Demographic and clinical data were collected, including sex, age, body mass index (BMI), systolic blood pressure (SBP), diastolic blood pressure (DBP), disease duration, medical history, clinical symptoms, vitamin D levels, QT interval, corrected QT interval (QTc), and QRS duration.

### 2.3 Scale assessments

The DHI is a symptom-specific scale that quantitatively evaluates the impact of dizziness across physical, functional, and emotional domains (13). It consists of 25 items grouped into four indices: total DHI score and three subscales (physical, emotional, and functional). Each item is scored as 4 (yes), 2 (sometimes), or 0 (no), with a maximum total score of 100 points. Higher scores indicate greater impairment in quality of life.

The 24-item Hamilton Depression Rating Scale (HAMD-24) was used to assess depressive symptoms, with scores positively correlated to depression severity: <7 (no depression), 7–17 (mild depression), 18–24 (moderate depression), and >24 (severe depression). The HAMD assessment should be conducted jointly by two trained evaluators. The evaluation typically involves a combination of conversation and observation. Upon its completion, the two evaluators should assign their scores independently (14).

The 14-item Hamilton Anxiety Rating Scale (HAMA-14) was used to assess anxiety symptoms. The 14 items of the HAMA are rated on a 5-point scale from 0 to 4, as follows: 0, absence of symptoms; 1, mild symptoms; 2, moderate symptoms (definite symptoms that do not interfere with daily life or activities); 3, severe symptoms (requiring treatment or already affecting daily activities); 4, extremely severe symptoms (severely impairing the patient’s life), with scores positively correlated to anxiety severity: <6 (no anxiety), 7–14 (possible anxiety), 15–21 (definite anxiety), 22–29 (marked anxiety), and ≥30 (severe anxiety) (15).

Sleep quality was measured using the Pittsburgh Sleep Quality Index (PSQI). The scale consists of seven major components: subjective sleep quality, sleep latency, sleep duration, sleep efficiency, sleep disturbances, sleep medication use, and daytime dysfunction. Each component is rated on a scale from 0 to 3, with the total score ranging from 0 to 21, with scores interpreted as: 0–5 (good sleep quality), 6–10 (mild sleep disturbance), 11–15 (moderate sleep disturbance), and 16–21 (severe sleep disturbance) (16).

The Somatic Self-Rating Scale (SSS) was used to assess the severity of somatic symptoms. The scale consists of 20 items, each rated on a 4-point scale without reverse scoring, as follows: 1, no symptoms; 2, mild symptoms; 3, moderate symptoms; and 4, severe symptoms, with total scores of >40 considered clinically significant (17).

### 2.4 Vestibular function tests

#### 2.4.1 Video head impulse test (VHIT)

Participants sat upright in a relaxed position and fixated on a target at eye level positioned 1–1.5 m ahead. The examiner applied small, rapid, passive, and abrupt impulses (amplitude: 10°–20°; peak head velocity >150°/s) in the plane of the horizontal semicircular canal.

For vertical semicircular canal assessment, two methods were employed: Method 1 involved rotating the head 45° to one side while fixating on an eye-level target, followed by application of impulses in the anteroposterior direction to evaluate the ipsilateral posterior and contralateral anterior canals. Method 2 was the same as Method 1 but with an additional calibration step before impulse testing. Each direction was tested 10–20 times. Normal gain values were: anterior/posterior canals, 0.7–1.2; and horizontal canal, 0.8–1.2 (18).

#### 2.4.2 Caloric test

The caloric test evaluates and compares the function of the bilateral horizontal semicircular canals by observing their responses to thermal stimulation. The examination is conducted in a darkened room. Prior to testing, the external auditory canal must be carefully inspected for the presence of cerumen, inflammation, injury, or tympanic membrane perforation to ensure thermal stimulation validity (19).

During the procedure, the individual lies in a supine position with the head elevated (flexed) at 30° to align the horizontal semicircular canal vertically. Subsequently, the patient is instructed to perform mental arithmetic to maintain alertness throughout eye movement recording. The stimulation is administered in the following sequence: right warm air, left warm air, right cold air, and left cold air. Eye movement recording begins 20 s prior to each air stimulus initiation. During the peak intensity of nystagmus (typically between 60 and 70 s after stimulus onset), a fixation light is activated, and the subject is asked to focus on the light for 10 s to evaluate fixation suppression. Eye movements are recorded until the nystagmus subsides or for a minimum of 2–3 min from stimulation start.

The interpretation of results is based on the analysis of the slow-phase velocity scatter diagram (butterfly diagram), in conjunction with the following parameters: (1) canal paresis, percentage difference between the sum and the difference of the slow-phase velocities of nystagmus elicited by stimulation of each ear (normal value, ≤25%); and (2) directional preponderance, percentage difference between the sum and the difference of the slow-phase velocities of left-beating and right-beating nystagmus (normal value ≤30%).

#### 2.4.3 Vestibular evoked myogenic potential (VEMP)

VEMPs are myogenic potentials elicited by strong acoustic or vibratory stimulation of the vestibular otolith organs, including cervical VEMP (cVEMP) from the sacculo-mediated sternocleidomastoid muscle and ocular VEMP (oVEMP) from the utricle-mediated extraocular muscle, which evaluate the function of the sacculo-inferior and utriculo-superior vestibular nerve pathways, respectively (18).

cVEMP testing: The reference electrode was placed on the sternoclavicular joint; the ground electrode on the forehead between the eyebrows; and the active electrodes on the upper third to half of the sternocleidomastoid muscles, with electrode impedance ≤5 kΩ. Stimuli included 500 Hz tone bursts (or 0.1 ms clicks) with rise/fall times of 1 ms, a plateau duration of 2 ms, a repetition rate of 5 Hz, and 50–100 averages. Recording parameters: time window 50 ms, bandpass filter 10–1, 000 Hz. Stimulus intensity started at 95–105 dB nHL or 115–130 dB SPL, decreasing in 5 dB steps until no identifiable VEMP waveform was detected. Participants were seated with maximal head rotation or supine with head elevated by 30° to maintain sternocleidomastoid muscle tension. Monoaural stimulation and ipsilateral recording were performed, alternating sides. Bilateral simultaneous stimulation/recording was also feasible in the supine head-elevated position.

oVEMP testing: Parameters were identical to cVEMP except 100 averages were used. The reference electrode was placed on the mandible; the ground electrode on the forehead between the eyebrows; and the active electrode 1 cm below the center of the contralateral lower eyelid. Participants were seated or supine, gazing upward at 25°–30° to maintain inferior oblique muscle tension while minimizing blinks. Monoaural stimulation and contralateral recording were performed, alternating sides. Bilateral simultaneous stimulation/recording was also feasible.

The following latencies can be observed (20): (1) cVEMP, the P1 latency (from stimulus onset to the P1 wave) is approximately 13 ms, and the N1 latency (from stimulus onset to the N1 wave) is approximately 23 ms; and (2) for oVEMP, the N1 latency (from stimulus onset to the N1 wave) is approximately 10 ms, and the P1 latency (from stimulus onset to the P1 wave) is approximately 15 ms.

### 2.5 Short-term HRV

Participants abstained from alcohol, coffee, strong tea, and strenuous activity for 12 h prior to testing. After resting for 15 min, HRV was measured with the participant in a supine position; with a test duration generally between 3 and 5 min. The testing room was kept at a constant temperature with minimal noise. Participants were asked to breathe normally; keep their eyes closed but remain awake; and refrain from coughing, silent reading, or mental arithmetic, as these could alter respiratory patterns (21).

The following parameters were assessed: Standard deviation of NN intervals (SDNN), normal range of 50–100 ms;low-frequency/high-frequency power ratio (LF/HF), normal range of 1.5–2.0.

### 2.6 Grouping method

A total of 144 patients with peripheral vestibular disorders were divided into four groups based on vestibular function test results: Group A, no abnormalities detected; Group B, abnormal otolith-related tests (cVEMP and oVEMP) but normal semicircular canal function; Group C, abnormal semicircular canal-related tests (VHIT and caloric test) but normal otolith function; and Group D, abnormalities in both semicircular canal- and otolith-related function tests. Group A had normal vestibular function tests; Group B had abnormal VEMP examination; Group C had CT and (or) V-HIT abnormalities; Group D had abnormal CT and (or) V-HIT and VEMP.

### 2.7 Statistical analysis

Statistical analyses were performed using SPSS version 27.0. Normally distributed data were expressed as mean ± standard deviation, while non-normally distributed data were presented as median and interquartile range. The Kolmogorov–Smirnov test was used to assess normality. One-way analysis of variance was applied for group comparisons of normally distributed data, while the Kruskal–Wallis or Mann–Whitney U tests were used for non-normally distributed data, as appropriate. Correlation analysis was conducted using Pearson’s correlation coefficient if both variables followed a bivariate normal distribution and exhibited a linear relationship. Spearman’s rank correlation was used if the assumptions for Pearson’s correlation were not met. Multiple linear regression was employed to identify independent influencing factors of HRV parameters. Statistical significance was set at a two-tailed *p* < 0.05.

## 3 Results

No significant differences were observed across the four groups in terms of sex; disease duration; BMI; SBP; DBP; and history of hypertension, diabetes, insomnia, anxiety, depression, and cardiovascular diseases (all *p* > 0.05). However, age and history of motion sickness showed significant differences between the groups (both *p* < 0.05; Table 1).

**Table 1:** Baseline characteristics of the four groups.

Baseline characteristic	A Group (*N* = 40)	B Group (*N* = 37)	C Group (*N* = 37)	D Group (*N* = 30)	*p*
Sex					
Male	12 (30.0%)	17 (45.9%)	14 (37.8%)	10 (33.3%)	0.516
Female	28 (70.0%)	20 (54.1%)	23 (62.2%)	20 (66.7%)	
Age	63.00 (48.25, 69.00)	66.00 (59.50, 70.00)	58.00 (39.00, 67.00)^a^	69.00 (56.00, 73.00)	0.003
Disease duration	730.00 (52.50, 1740.25)	750.00 (67.50, 3520.50)	730.00 (105.00, 1825.00)	639.00 (82.50, 1551.25)	0.880
BMI (kg/m^2^)	25.19 ± 3.74	24.01 ± 4.36	24.93 ± 3.21	24.02 ± 2.44	0.365
SBP (mmHg)	137.61 ± 18.52	137.17 ± 17.76	128.97 ± 15.17	137.60 ± 15.90	0.645
DBP (mmHg)	88.54 ± 14.87	87.00 ± 11.46	83.50 ± 11.16	82.53 ± 9.62	0.385
Hypertension					
Yes	23 (57.5%)	16 (43.2%)	13 (35.1%)	17 (56.7%)	0.164
No	17 (42.5%)	21 (56.8%)	24 (64.9%)	13 (43.3%)	
Diabetes					
Yes	8 (20.0%)	6 (16.2%)	5 (13.5%)	6 (20.0%)	0.861
No	32 (80.0%)	31 (83.8%)	32 (86.5%)	24 (80.0%)	
Insomnia					
Yes	6 (15.0%)	8 (21.6%)	3 (8.0%)	5 (16.7%)	0.449
No	34 (85.0%)	29 (78.4%)	34 (92.0%)	25 (83.3%)	
Anxiety depression					
Yes	1 (2.5%)	6 (16.2%)	4 (10.8%)	9 (30.0%)	0.156
No	39 (97.5%)	31 (83.8%)	33 (89.2%)	21 (70.0%)	
Heart disease					
Yes	8 (20.0%)	4 (10.8%)	7 (18.7%)	5 (16.7%)	0.715
No	32 (80.0%)	33 (89.2%)	30 (81.3%)	25 (83.3%)	
Carsickness					
Yes	12 (30.0%)^a^	14 (37.8%)	11 (29.7%)^a^	19 (63.3%)	0.018
No	28 (70.0%)	23 (62.2%)	26 (70.3%)	11 (39.7%)	

a Compared with group D, *p* < 0.05.

BMI, body mass index; SBP, systolic blood pressure; DBP, diastolic blood pressure.

No significant differences were observed across the four groups in terms of position-related dizziness, headache, nausea, vomiting, tinnitus, ear fullness, hearing loss, diplopia, blurred vision, palpitations, and limb weakness (all *p* > 0.05). However, photophobia, phonophobia, and snoring showed significant intergroup differences (all *p* < 0.05; Table 2).

**Table 2:** Comparison of clinical symptoms across the four groups.

Concomitant symptoms	A Group (*N* = 40)	B Group (*N* = 37)	C Group (*N* = 37)	D Group (*N* = 30)	*p*
Position-related dizziness					
Yes	16.0 (40.00%)	16.00 (43.20%)	18 (48.65%)	9.00 (24.32%)	0.480
No	24.00 (60.00%)	21.00 (56.80%)	19 (51.35%)	21.00 (75.68%)	
Headache					
Yes	13.00 (32.50%)	10.00 (27.03%)	11.00 (29.73%)	7.00 (23.33%)	0.857
No	27.00 (67.50%)	27.00 (72.97%)	26.00 (70.27%)	23.00 (76.67%)	
Nausea					
Yes	20.00 (50.00%)	16.00 (43.20%)	24.00 (64.86%)	12.00 (40.00%)	0.164
No	20.00 (50.00%)	21.00 (56.80%)	13.00 (35.14%)	18.00 (60.00%)	
Vomiting					
Yes	16 (40.00%)	10.00 (27.03%)	17.00 (45.95%)	11.00 (36.67%)	0.399
No	24 (60.00%)	27.00 (72.97%)	20.00 (54.05%)	19.00 (63.33%)	
Tinnitus					
Yes	11.00 (27.50%)	14.00 (37.84%)	21.00 (56.76%)	11.00 (36.67%)	0.068
No	29.00 (62.50%)	23.00 (62.16%)	16.00 (43.24%)	19.00 (63.33%)	
Ear fullness					
Yes	4.00 (10.00%)	1.00 (2.70%)	5.00 (13.51%)	6.00 (20.00%)	0.153
No	36.00 (90.00%)	26.00 (97.30%)	32.00 (86.49%)	24.00 (80.00%)	
Hearing loss					
Yes	2.00 (5.00%)	8.00 (21.62%)	11.00 (29.73%)	9.00 (30.00%)	0.364
No	38.00 (95.00%)	29.00 (78.38%)	26.00 (70.27%)	21.00 (70.00%)	
Photophobia					
Yes	4.00 (10.00%)^a^	3.00 (8.11%)^a^	13.00 (35.14%)	4.00 (13.33%)	0.006
No	36.00 (90.00%)	34.00 (91.89%)	24.00 (64.86%)	26.00 (86.67%)	
Phonophobia					
Yes	0.00 (0.00%)^a^	1.00 (2.70%)	8.00 (21.62%)	1.00 (3.33%)^a^	<0.001
No	40.00 (100.00%)	26.00 (97.30%)	29.00 (78.38%)	29.00 (96.67%)	
Diplopia					
Yes	0.00 (0.00%)	1.00 (2.70%)	0.00 (0.00%)	1.00 (3.33%)	0.506
No	40.00 (100.00%)	26.00 (97.30%)	37.00 (100.00%)	29.00 (96.67%)	
Blurred vision					
Yes	5.00 (12.50%)	6.00 (16.22%)	2.00 (5.41%)	4.00 (13.33%)	0.552
No	35.00 (87.50%)	31.00 (83.78%)	35.00 (94.59%)	26.00 (86.67%)	
Palpitations					
Yes	8.00 (20.00%)	5.00 (13.51%)	7.00 (18.92%)	5.00 (16.67%)	0.877
No	32.00 (80.00%)	32.00 (86.49%)	30.00 (81.08%)	25.00 (83.33%)	
Snoring					
Yes	4.00 (10.00%)^b^	16.00 (43.20%)	7.00 (18.92%)	6.00 (20.00%)	0.006
No	36.00 (90.00%)	21.00 (56.80%)	30.00 (81.08%)	24.00 (80.00%)	
Limb weakness					
Yes	11.00 (27.50%)	13.00 (35.14%)	14.00 (37.84%)	11.00 (36.67%)	0.776
No	29.00 (62.50%)	24.00 (64.86%)	23.00 (62.16%)	19.00 (63.33%)	

a Compared with group C, *p* < 0.05.

b Compared with group B, *p* < 0.05.

No significant differences were observed across the four groups in terms of the DHI-emotional, HAMD, HAMA, and SSS scores (all *p* > 0.05). However, the DHI-total, DHI-physical, DHI-functional, PSQI, and sleep efficiency scores showed significant differences between the groups (all *p* < 0.05; Table 3).

**Table 3:** Comparison of scale scores across the four groups.

Scale scores	A Group (*N* = 40)	B Group (*N* = 37)	C Group (*N* = 37)	D Group (*N* = 30)	*p*
DHI	30.00 (20.00, 34.00)^a^	30.00 (25.00, 42.00)^a^	36.00 (29.00, 41.00)	46.00 (34.00, 51.00)	<0.001
DHI-physical	8.00 (6.00, 10.00)^a^	12.00 (8.00, 16.00)^b^	10.00 (6.00, 12.00)^a^	14.00 (9.00, 19.00)	<0.001
DHI-emotional	10.00 (6.00, 14.00)	8.00 (6.00, 14.00)	10.00 (7.00, 16.00)^b^	12.00 (8.00, 16.00)	0.179
DHI-functional	8.00 (6.00, 14.00)^a^	12.00 (8.00, 17.00)	14.00 (9.00, 20.00)	18.00 (11.00, 26.00)	<0.001
Sleep efficiency	1.60 ± 1.13	1.83 ± 1.13	1.22 ± 1.22^a^	2.20 ± 1.16	0.008
PSQI	9.00 (6.00, 11.75)	10.00 (6.00, 14.75)	6.50 (4.00, 12.00)^a^	13.00 (7.50, 15.00)	0.002
>5	31 (77.50%)	29 (78.34%)	20 (54.05%)	27 (90.00%)	
HAMD	13.00 (9.00, 16.00)	12.00 (8.00, 17.00)	11.00 (8.00, 16.00)	14.50 (8.00, 20.00)	0.270
≤7	6 (15.00%)	8 (21.62%)	8 (21.62%)	4 (13.33%)	
>7	34 (85.00%)	29 (78.38%)	29 (78.38%)	26 (86.67%)	
HAMA	9.00 (6.25, 11.00)	10.00 (8.00, 13.50)	9.00 (6.00, 12.50)	11.00 (7.00, 14.25)	0.127
≤7	16 (40.00%)	7 (18.92%)	15 (40.54%)	10 (33.33%)	
>7	24 (60.00%)	30 (81.08%)	22 (59.46%)	20 (66.67%)	
SSS	37.50 (32.50, 45.00)	40.00 (33.50, 49.50)	43.00 (36.50, 45.50)	40.00 (34.25, 55.00)	0.389

a Compared with Group D, *p* < 0.05.

b Compared with Group A, *p* < 0.05.

DHI, dizziness handicap inventory; PSQI, Pittsburgh sleep quality index; HAMA, 14-item Hamilton anxiety rating scale; HAMD, 24-item Hamilton depression rating scale; SSS, somatic self-rating scale.

No significant differences were observed across the four groups in terms of LF/HF ratio, SDNN, QT interval, QTc interval, and QRS duration (all *p* > 0.05). However, RRIV showed a significant difference between the groups (*p* < 0.05; Table 4).

**Table 4:** Comparison of short-term HRV parameters across the four groups.

HRV and EEG	A Group (*N* = 40)	B Group (*N* = 37)	C Group (*N* = 37)	D Group (*N* = 30)	*p*
LH/HF	1.49 (0.83, 3.02)	1.81 (1.17, 2.83)	1.40 (0.93, 2.76)	1.29 (0.85, 2.57)	0.636
SDNN (ms)	36.95 (24.05, 42.83)	26.60 (22.60, 35.45)	34.70 (22.95, 47.50)	27.00 (18.50, 38.40)	0.190
QT	412.00 (385.00, 430.50)	400.00 (375.50, 430.50)	400.00 (381.00, 426.00)	398.00 (370.00, 422.00)	0.417
QTc	438.50 (419.75, 459.50)	440.00 (430.25, 465.25)	443.00 (411.00, 460.00)	434.00 (425.00, 445.00)	0.414
QRS duration	96.00 (88.00, 100.50)	95.00 (88.00, 102.50)	96.00 (92.00, 101.00)	96.00 (87.00, 100.00)	0.644

SDNN, standard deviation of NN intervals; LF/HF, low-frequency/high-frequency power ratio.

Spearman’s correlation analysis revealed that the PSQI (*r* = 0.203, *p* = 0.017), HAMD (*r* = 0.303, *p* < 0.001), and HAMA (*r* = 0.210, *p* = 0.014) scores were positively correlated with the DHI scores (all *p* < 0.05). No significant correlations were found between the DHI and age, BMI, disease duration, or vitamin D levels (Table 5).

**Table 5:** Correlation analysis between DHI scores and demographic/scale variables.

	*r*	*p*
Age	0.112	0.189
BMI	0.148	0.086
Disease duration	0.029	0.734
PSQI	0.203	0.017
HAMD	0.303	<0.001
HAMA	0.210	0.014
Vitamin D	−0.146	0.121

BMI, body mass index; DHI, dizziness handicap inventory; PSQI, Pittsburgh sleep quality index; HAMD, Hamilton depression scale (Hamilton depression rating scale); HAMA, Hamilton anxiety scale (Hamilton anxiety rating scale).

SDNN revealed negative correlations with DHI (*r* = −0.267, *p* = 0.002), HAMD (*r* = −0.167, *p* = 0.046), and QTc interval (*r* = −0.280, *p* = 0.001). A trend toward negative correlation was observed with PSQI (*r* = −0.156, *p* = 0.063), though this did not reach statistical significance. No significant associations were found between SDNN and age, BMI, disease duration, HAMA, or vitamin D levels (Table 6).

**Table 6:** Correlation analysis between SDNN and demographic variables.

	*r*	*p*
Age	−0.233	0.005
BMI	−0.143	0.091
Disease duration	−0.063	0.453
DHI	−0.267	0.002
PSQI	−0.156	0.063
HAMD	−0.167	0.046
HAHA	0.008	0.922
Vitamin D	0.157	0.086
QTc	−0.280	0.001

BMI, body mass index; DHI, dizziness handicap inventory; PSQI, Pittsburgh sleep quality index; HAMD, Hamilton depression rating scale; HAMA, Hamilton anxiety scale; QTc, corrected QT interval; SDNN, standard deviation of normal-to-normal intervals.

Multivariate linear regression using SDNN as the dependent variable identified the DHI score as an independent influencing factor (*p* < 0.05), after adjusting for age, HAMD, and QTc interval (Table 7).

**Table 7:** Multiple linear regression analysis identifying predictors of SDNN.

	*β*	SE	*t*	95% CI	*p*
Intercept	−0.108	0.475	−0.227	−1.047–0.832	0.821
Age	0.003	0.007	0.501	−0.010–0.017	0.618
DHI	0.034	0.007	4.893	0.020–0.048	<0.001
HAMD	−0.001	0.018	−0.042	−0.037–0.036	0.967
QTc	0.000	0.069	0.831	0.000–0.001	0.408

DHI, dizziness handicap inventory; HAMD, Hamilton depression rating scale, QTc, Corrected QT interval; SDNN, standard deviation of normal-to-normal intervals; SE, standard error; CI, confidence interval.

## 4 Discussion

### 4.1 Emotional correlates of vestibular dysfunction

Our study demonstrated significant positive correlations between DHI scores and HAMD, HAMA, and PSQI scores, indicating that higher emotional distress and poorer sleep quality were associated with greater perceived vestibular handicap. Notably, these results are consistent with those reported by Lindell et al. (22). Moreover, corroborating the findings by Whitney et al. (23)^,^ we found that elevated DHI scores, particularly in Group D, reflected more severe functional impairment, likely due to multi-site vestibular lesions. The comparable DHI scores between Groups B and C suggest that the DHI cannot differentiate single-site lesions nor reliably assess the severity of peripheral vestibular deficits, supporting the conclusions of Yip et al. (24).

The vestibular cortex integrates vestibular, visual, proprioceptive, somatosensory, and motor information and shares significant overlap with limbic structures regulating anxiety and emotional responses, particularly the posterior parietal cortex, hippocampus, and prefrontal regions (25). Structural and functional connections between the vestibular system and affective, cognitive, and autonomic networks are mediated by three principal pathways: thalamocortical projections (26), vestibulocerebellar circuits (27), and monoaminergic neurotransmitter systems (28). In peripheral vestibular damage, emotional disturbances are primarily modulated via three distinct neural pathways: vestibular nucleus-parabrachial nucleus-amygdala (29), vestibular nucleus-dorsal raphe nucleus, and vestibular nucleus-locus coeruleus (30). Vestibular dysfunction may lead to abnormal activity in these brain regions, thereby triggering emotional disorders such as anxiety and depression. Conversely, anxiety may also exacerbate dizziness perceptions.

All groups demonstrated elevated rates of anxiety and depression, with depression more prevalent than anxiety, in contrast to previous epidemiological reports (31, 32). The emotional effects of vestibular dysfunction appear to stem not only from the organic vestibular lesion, but also from psychological factors that play a crucial role in the development of anxiety and depression among patients with vertigo (33–35). This neuropsychological interplay was further elucidated using the Hilber’s Internal Model hypothesis (26). Clinical evidence indicates that anxiety severity correlates more with vertigo intensity than with specific vestibular pathology or underlying pathophysiological mechanisms (36). This explains the comparable HAMA and HAMD scores across our study groups and supports the view that the anxiety-depression-vestibular relationship is bidirectional. Affective disorders can reciprocally influence vestibular responsiveness by modulating caloric test parameters, including response duration, directional preponderance, and slow-phase velocity of vestibulo-ocular reflexes (37, 38).

### 4.2 Sleep–vestibular interactions

Sleep regulates and consolidates neural activity in visual, auditory, and olfactory cortices, a process similar to that observed in the hippocampus (39). Multiple neural pathways, such as the vestibulo-cerebello-cortical, vestibulo-thalamo-cortical, and head-direction pathways, connect the vestibular system to the hippocampus (40). Considering the role of sleep in neuroplasticity and the influence of the vestibular system on hippocampal function, normal sleep may facilitate vestibular neural circuit repair, support vestibular system functional recovery, and contribute to its regulatory processes, potentially facilitating the integration of this information into vestibular and/or multisensory cortical regions (41). Anatomical connections between the vestibular nuclei and hypothalamic regions enable vestibular inputs in wake–sleep regulation (42). However, the precise mechanisms through which sleep disturbances affect vestibular function remain controversial. While some studies report impaired vestibulo-ocular reflexes following sleep deprivation, others show no significant vestibular changes. Our data reveal significantly higher PSQI scores in Groups D compared to that in Group C, suggesting a key role of otolithic (utricular and saccular) dysfunction in sleep disturbances. As our study did not include VEMP subgroup analyses, further investigation is needed to clarify specific otolithic contributions. Lin et al. (43) reported higher oVEMP abnormalities in sleep-deprived individuals, possibly due to altered spatial processing in the posterior parietal cortex, which affects vestibular integration (44). Some researchers propose that the sleep efficiency metrics in the PSQI may indirectly reflect sleep deprivation (45). Our finding of higher sleep efficiency scores in Groups B and D supports the role of otolithic dysfunction in sleep-deprived patients. A recent review highlighted that sleep disorders may impair neuroplasticity-mediated vestibular compensation, underscoring the need for combined vestibular rehabilitation and sleep management in therapeutic strategies.

Obstructive sleep apnea (OSA), characterized by intermittent hypoxia from recurrent breathing interruptions, can induce vascular endothelial damage and inflammation affecting both vestibular end organs and central pathways (46). Xu et al. (47) reported a high prevalence of vertigo/dizziness among 512 patients with OSA. Although VEMP abnormalities have been found in patients with OSA, these appear to be independent of OSA severity (48, 49). Habitual snoring, a diagnostic criterion of OSA (50)^,^ was more prevalent in Group B, suggesting possible OSA-related nocturnal cerebral hypoxemia. Given the richer vascular supply to the semicircular canals compared to the otolithic organs, this hypoxic state might preferentially affect otolithic function. Future polysomnographic studies are warranted to elucidate this potential association.

### 4.3 Autonomic regulation and HRV

Vestibular autonomic reflexes, which influence sympathetic and parasympathetic efferent pathways through multiple routes (51)^,^ are mediated via descending projections from the medial and inferior vestibular nuclei to the dorsal motor nucleus of the vagus and medullary nucleus, and to the ascending fibers from the superior vestibular nucleus to the parabrachial complex (52). Vestibular lesions in these areas may disrupt autonomic regulation, contributing to symptoms such as nausea and vomiting (53). SDNN, a marker of overall autonomic regulation (7), was <50 ms in all groups, indicating sympathetic dominance and parasympathetic suppression in patients with vertigo/dizziness. Reportedly, sympathetic excitation increases catecholamine levels, which may interact with vestibular function through pathways involving the amygdala and hypothalamus (54, 55). Although central compensatory mechanisms may activate parasympathetic pathways (56), in this study, the impaired autonomic regulation of the patients likely prevented adequate parasympathetic activation, resulting in sympathetic dominance and related symptoms.

Although factors such as mood can influence SDNN (57), the absence of significant differences in the HAMA/HAMD scores across groups suggests that emotional states did not affect the SDNN results. A negative correlation between the DHI scores and SDNN were observed, with DHI being an independent contributing factor to reduced SDNN. Specifically, for every 1-point increase in the DHI score, SDNN decreased by 0.034 ms, suggesting that dizziness severity may be associated with worsening autonomic dysfunction, although the possibility of reverse causality, wherein autonomic dysfunction exacerbates dizziness, cannot be ruled out. Group D showed the highest DHI scores and lowest SDNN values across the groups, indicating that more extensive vestibular involvement may correspond to greater autonomic impairment, possibly due to insufficient central vestibular compensation.

### 4.4 Age-related effects

Vestibular hair cell and neuronal degeneration begins between ages 20 and 40 (58). We observed a negative correlation between age and SDNN, indicating a decline in autonomic regulatory capacity with aging, which is consistent with the findings of Klassen et al. (59).

## 5 Clinical implications and future directions

The present study confirmed a close association between multi-site vestibular impairment and clinical symptom severity, indicating that patients with combined dysfunction of both the semicircular canals and otolith organs (Group D) exhibited significantly worse outcomes in DHI total score, DHI physical subscore, DHI functional subscore, and PSQI compared to those with normal vestibular function or isolated damage to a single site. Therefore, multi-site vestibular lesions may lead to more severe functional impairment and autonomic dysfunction, highlighting the need for increased clinical attention and comprehensive evaluation in this patient population.

Furthermore, this study established an objective link between dizziness severity and autonomic dysregulation: DHI scores were independently and negatively correlated with SDNN, indicating that worse subjective dizziness is associated with poorer autonomic nervous system regulation, providing a practical basis for using the DHI scale as a preliminary tool to assess autonomic status in clinical settings where HRV measurement is not available.

Additionally, this study clarified the interactions among vestibular function, sleep, and emotional state. Although statistically significant, the positive correlations between DHI and PSQI, HAMD, and HAMA were relatively weak, suggesting that dizziness, sleep disturbances, and emotional distress frequently co-occur and may mutually reinforce one another. Notably, otolithic dysfunction appeared to be more strongly associated with impaired sleep efficiency, offering a new perspective on the mechanisms underlying sleep disorders in patients with vestibular pathologies.

Future research should further expand the sample size and stratify by etiology to control for confounding factors; incorporate objective measures such as polysomnography and neuroimaging to explore the causal relationship and neural mechanisms between otolith dysfunction and sleep disorders; conduct longitudinal studies to track changes in autonomic nervous function during vestibular compensation; and develop comprehensive intervention strategies for patients with multi-site vestibular damage based on these findings, evaluating their effects on vertigo symptoms, emotional state, and sleep quality.

## Data Availability

The raw data supporting the conclusions of this article will be made available by the authors, without undue reservation.
